# “Crossing borders, connecting cultures”: an introduction to the special issue

**DOI:** 10.1186/s40878-023-00333-4

**Published:** 2023-03-23

**Authors:** Birte Nienaber, Nicole Holzapfel-Mantin, Gabriele Budach

**Affiliations:** Faculty of Humanities, Education and Social Sciences, Université du Luxembourg, Esch-sur-Alzette, Luxembourg

## Abstract

This special issue of *Comparative Migration Studies* on the occasion of the IMISCOE 2021 Conference with the theme “Crossing borders, connecting cultures” features five invited contributions by several conference speakers as well as an article by the host university.

This special issue of *Comparative Migration Studies* on the occasion of the IMISCOE 2021 Conference with the theme “Crossing borders, connecting cultures” features invited contributions by several conference speakers as well as an article by the host university. This conference was planned to take place in July of 2020 at the University of Luxembourg and ultimately had to be postponed to 2021, when it took place online. The reason for the postponement of the conference, the Covid-19 pandemic, did significantly influence the papers presented at the conference and it also changed the way this conference’s theme of “Crossing borders, connecting cultures” was perceived. This also holds true for the invited speakers who contributed articles to this issue. They come from very different backgrounds—in terms of their disciplinary affiliation, the topics they presented on and the geographical environments they fell attached to, live and work in. We would like to briefly re-trace for you this remarkable journey during which different borders were crossed and cultures connected across disciplinary traditions and boundaries.

Previous conferences have looked into migration, the political, and economic forces driving it, the ways it is patterned, administered and controlled by state border regimes, and the consequences migration has created for people who move and societies accommodating people on the move. From this point of departure, we originally intended to shift the focus more to people’s migration experiences by foregrounding how migration is connected to culture. We wanted to explore the nexus of migration and culture in more depth, asking how migration is lived, experienced, reflected and mediated, in particular through cultural and artistic practice.

However, by the time we started planning our rescheduled conference in early 2021, a lot had changed in a very short period of time. Not only did we feel the direct impact of the pandemic on our health or the health of those close to us, we were also just beginning to grapple with the societal impacts of and the responses to this pandemic.

One of many impacts felt was the COVID-19-related border closures, which limited free movement and thus have shaken the foundation of the European Union. Yet it was not only in Europe that border closures took effect. Many migrants, as well as international students, were and are affected profoundly, as they cannot/could not return and hence were/are trapped in their destination countries—often without any income. Since many migrants work in critical sectors such as the health care sector, the important role they play in the response to the crisis increased their exposure to the virus. Data from New York showed that due to income inequality and marginalisation, migrants were overrepresented in neighbourhoods that were most affected by the virus. Further, this pandemic made existing inequalities more visible and it amplified them, as the strong and worldwide Black Lives Matter protests showed.

Reflecting on these significant developments, we needed to reassess the meaning of “Crossing borders, connecting cultures.” We decided to extend our focus to include contributions on inequalities in general and on health and racial inequalities in particular. This special issue brings together approaches originating in multiple research fields which discuss the manifold ways in which boundaries can be crossed and cultures can be connected. The results are illuminating insights into how people’s experience of migration is connected to culture, as well as many new connections that extend across disciplinary boundaries.

At the time of writing this introduction, in spring of 2023, Covid-19 seems to have become controllable and life seems to be returning to more or less pre-pandemic ways. Yet in light of the current crisis in Ukraine, we ask ourselves how the meaning of crossing borders and connecting cultures will need to be rethought once again, and what role the field of migration studies, including research on arts and culture, will play—a question which needs addressing in future discussions and exchanges.

This collection of *five* articles has successfully spanned the gamut, with contributions from very different fields such as anthropology, sociology, cultural psychology, behavioural and cognitive sciences and linguistics, to theory, arts and culture, borders and border experiences, to health and racial inequalities in migration research. As in the conference, these papers have reached across disciplinary boundaries not only in terms of their content but also in how they were written.

In the paper “*We are all migrants*”, *Jaan Valsiner* examines, from the perspective of cultural psychology of semiotic dynamics, the process of becoming and being a “migrant” or “counter-migrant” by looking at the relation between the people who become migrants and those who do not.

Jaan Valsiner pioneered the introduction of the concept of semiotic dynamics to cultural psychology. In cultural psychology, this concept assumes that humans are “constantly creating, maintaining and abandoning hierarchies of meanings within all cultural contexts they experience.” By applying this idea to the field of migration research, he is able to demonstrate how societies are trapped in a system of social representations of people who become migrants created by the people who are not migrants, or the “counter-migrants”. He underlines his line of argument with an example in which a community moves to a different location where nobody lives and which would be referred to as a resettlement. However, if this community were to move to a spot that is already populated, this process would be called migration, and would most likely entail several issues. Hence, he argues that we need to take a closer look at the “counter-migrants” who create assumptions of migrants because they see them as the “unknown other”. Within this relation, which can be anything between positive to negative and which is often renegotiated, invisible borders are constantly crossed. He ends by referring back to his opening statement: “Migration is the basis for development—economic, social, and psychological” and concludes that in a world without migration, we would live in closed systems without any exchanges. Such exchanges, however, are indispensable for our societies to flourish and develop, which is why “we will always be migrants.” When borders are crossed but connections of any kind are unwanted, unfavourable situations are created for migrants. This situation is aggravated when borders are suddenly closed and migrants cannot move back to either their home countries or their destination countries.

In her article “Towards equality: joining forces with arts and culture in the struggle for change in migration societies”, *Wiebke Sievers* illustrates how migration is being connected to culture and elaborates why migration research needs to research artistic and cultural practices of immigrants and their descendants. She uses the example of the work of the Palestinian–Danish poet Yahya Hassan to illustrate that migrant art and culture can open up new perspectives by allowing personal insights into the lives of migrants, which can lead to a change in how migrants are perceived. Hence, artistic and cultural activities by migrants can be helpful for moving beyond the idea of homogeneous national identities and cultures, by rethinking communities. Since migration researchers and other humanities scholars both strive towards equality, the author argues for the crossing of disciplinary boundaries. Once these boundaries are crossed, mutual understanding could eventually allow artistic and cultural activities of migrants to be seen as demands for equality. Wiebke Sievers argues that including migrant art and culture has the potential to change how we see the world, which in turn affects how we conceive of not only migration and migrants, but also community and social justice. Art can help with “imagining new narratives of society.”

Cultures are being connected and borders are crossed in many different ways and over time, as *Marco Martiniello* demonstrates in his paper “*Researching arts, culture, migration and change: a multi (trans)disciplinary challenge for international migration studies.*” In a way similar to Wiebke Sievers’ contribution, the author points out that in migration research, the links between arts, culture and migration and their significance are often overlooked.

Marco Martiniello begins by providing a brief historical overview to illustrate why migrants’ arts and cultures have not been recognised and are still not fully recognised in society and academia. He goes on to build arguments about why migration research needs to pay attention to and reflect on the links between migration, arts and cultures, which have always existed despite having been ignored. Through two case studies concerning a Senegalese painter and sculptor in Brussels and an action-theatre company in the Liège region, he demonstrates not only that these links exist, but also how significant they are. Art provides plenty of opportunities for migrants to communicate stories of migrations and to raise awareness of the dangers and difficulties many migrants face on their way to Europe and within their host countries, and to express related emotions. Hence, together, arts and culture contribute to identity construction. The audience will recognise that “Migrants are not only muscles and arms, workers, and factors of production, but also agents of artistic and cultural change.” Hence, migrant art can make an immense contribution to social cohesion. Seeing the potential of art and culture in migration research, Marco Martiniello suggests that migration research should cross disciplinary borders and connect disciplinary cultures to enable us to understand these connections.

How disciplinary borders can be crossed to create new insights into connecting cultures is shown in the paper “*Migration and conviviality: Living with difference in Luxembourg.*” A group of authors from very different disciplinary backgrounds (*Elisabeth Boesen, Gabriele Budach, Isabelle Albert, Elke Murdock, Birte Nienaber, Stephanie Barros, Stéphanie Delgado, Melany Navalha, Marc Campill*) worked together to gain a truly interdisciplinary view on diversity and ways of ‘living together’ in the conference’s host country of Luxembourg, to comprehend how difference is constructed, experienced and negotiated, with a focus on day-to-day situations. This article consists of two parts, each of them representing a different lens of observation and analysis. The first part looks into language and multilingualism and the complex ways in which language contributes to creating social relations of difference, notably in the workplace, but also in other contexts of convivial living. This part includes the views of Luxembourgish mothers and their adult children, on young professionals, mostly from EU countries, who move to Luxembourg for work, and on Japanese women in Luxembourg, The lens on language and multilingualism foregrounds a highly visible dimension in Luxembourgish society, while the second part of the paper deals with much less visible phenomena—relating to religious and cultural practices and forms of racial discrimination that only begin to receive attention in public discourse and policy. This part includes research work conducted on and with Cape Verdean and Portuguese migrants: (1) on practices of mourning of as a specific domain of ceremonial expression and (2) on film making as a medium of self-reflection and visualisation. This section centres on aspects of diversity that are invisible in Luxembourg, namely on non-European immigration and emerging encounters with difference, hypothesising that visible minorities are treated as non-existent, and experience themselves as being treated thus. The authors draw on the strength resulting from the cooperation of several disciplines to synthesise the insights they have gained and to connect these with the notion of conviviality. This, in turn, enables them to flesh out the relation(s) between mundane encounters and persisting power inequalities and conflict.

The paper by *Dudziro Nhengu* on “*Covid‑19 and female migrants: policy challenges and multiple vulnerabilities*” looks at how the pandemic has further worsened the situation of many female economic migrants in South Africa, and therefore forms a bridge to the Global South as well as to the migration-culture nexus during the pandemic. The majority of female migrants in South Africa work in the (health) care sector, where they experience high exposure (not only) to the Covid-19 virus. Many female migrants are not documented in their destination country, which leaves them without protection of their workers’ rights, and their rights to intact sexual and reproductive health. Against this background, the author approaches her question though the lens of vulnerability of female, mostly irregular, migrants. Together with her case study, which includes strategic conversations with 15 Zimbabwean women working in South Africa and Botswana, her perspective enables her to reveal that existing policies are often gender-blind, and how they contributed to the further deterioration of the situation of many migrant women. Dudziro Nhengu goes on to argue that gender-sensitive migration policies and practices are necessary in order respond to the “multifaceted and complex relationship between migration, Covid-19 policy and economic development” as well as the situation of female migrants.

## Data Availability

Not applicable.

